# Gelatinase-A (MMP-2), gelatinase-B (MMP-9) and membrane type matrix metalloproteinase-1 (MT1-MMP) are involved in different aspects of the pathophysiology of malignant gliomas

**DOI:** 10.1038/sj.bjc.6990291

**Published:** 1999-04

**Authors:** P A Forsyth, H Wong, T D Laing, N B Rewcastle, D G Morris, H Muzik, K J Leco, R N Johnston, P M A Brasher, G Sutherland, D R Edwards

**Affiliations:** Departments of Clinical Neurosciences and Medicine, Epidemiology and Preventive Oncology, Tom Baker Cancer Centre, 1331 29 St NW, Calgary, Alberta, T2N 4N2, Canada; Departments of Medical Biochemistry, Clinical Neurosciences, Community Health Sciences, The University of Calgary, 3330 Hospital Drive NW, Calgary, Alberta, T2N 4N1, Canada; Departments of Pathology and Clinical Neurosciences, Foothills Hospital, Calgary, Alberta, T2N 4N1, Canada

**Keywords:** gliomas, gelatinase-A, gelatinase-B, MT1-MMP, in situ hybridization

## Abstract

Matrix metalloproteinases (MMPs) have been implicated as important factors in gliomas since they may both facilitate invasion into the surrounding brain and participate in neovascularization. We have tested the hypothesis that deregulated expression of gelatinase-A or B, or an activator of gelatinase-A, MT1-MMP, may contribute directly to human gliomas by quantifying the expression of these MMPs in 46 brain tumour specimens and seven control tissues. Quantitative RT-PCR and gelatin zymography showed that gelatinase-A in glioma specimens was higher than in normal tissue; these were significantly elevated in low grade gliomas and remained elevated in GBMs. Gelatinase-B transcript and activity levels were also higher than in normal brain and more strongly correlated with tumour grade. We did not see a close relationship between the levels of expression of MT1-MMP mRNA and amounts of activated gelatinase-A. In situ hybridization localized gelatinase-A and MT1-MMP transcripts to normal neuronal and glia, malignant glioma cells and blood vessels. In contrast, gelatinase-B showed a more restricted pattern of expression; it was strongly expressed in blood vessels at proliferating margins, as well as tumour cells in some cases. These data suggest that gelatinase-A, -B and MT1-MMP are important in the pathophysiology of human gliomas. The primary role of gelatinase-B may lie in remodelling associated with neovascularization, whereas gelatinase-A and MT1-MMP may be involved in both glial invasion and angiogenesis. © 1999 Cancer Research Campaign


British Journal of Cancer (1999) 79(11/12), 1828–1835
© 1999 Cancer Research Campaign
Article no. bjoc.1998.0291

### Gelatinase-A (MMP-2), gelatinase-B (MMP-9) and membrane type matrix metalloproteinase-1 (MT1-MMP) are involved in different aspects of the pathophysiology of malignant gliomas

**PA Forsyth[1,4,6], H Wong[3], TD Laing[3], NB Rewcastle[6], DG Morris[1,4,6], H Muzik[1,3,4,6], KJ Leco[3,*], RN Johnston[3], PMA Brasher[2], G Sutherland[7] and DR Edwards[3]**

Departments of [1]Clinical Neurosciences and Medicine, and [2] Epidemiology and Preventive Oncology, Tom Baker Cancer Centre, 1331 29 St NW, Calgary, Alberta, Canada T2N 4N2; Departments of [3]Medical Biochemistry, [4]Clinical Neurosciences and [5]Community Health Sciences, The University of Calgary, 3330 Hospital Drive NW, Calgary, Alberta, Canada T2N 4N1; Departments of [6]Pathology and Clinical Neurosciences, Foothills Hospital Calgary, Alberta, Canada, T2N 4N1

**Summary** Matrix metalloproteinases (MMPs) have been implicated as important factors in gliomas since they may both facilitate invasion into the surrounding brain and participate in neovascularization. We have tested the hypothesis that deregulated expression of gelatinase-A or B, or an activator of gelatinase-A, MT1-MMP, may contribute directly to human gliomas by quantifying the expression of these MMPs in 46 brain tumour specimens and seven control tissues. Quantitative RT-PCR and gelatin zymography showed that gelatinase-A in glioma specimens was higher than in normal tissue; these were significantly elevated in low grade gliomas and remained elevated in GBMs. Gelatinase-B transcript and activity levels were also higher than in normal brain and more strongly correlated with tumour grade. We did not see a close relationship between the levels of expression of MT1-MMP mRNA and amounts of activated gelatinase-A. In situ hybridization localized gelatinase-A and MT1-MMP transcripts to normal neuronal and glia, malignant glioma cells and blood vessels. In contrast, gelatinase-B showed a more restricted pattern of expression; it was strongly expressed in blood vessels at proliferating margins, as well as tumour cells in some cases. These data suggest that gelatinase-A, -B and MT1-MMP are important in the pathophysiology of human gliomas. The primary role of gelatinase-B may lie in remodelling associated with neovascularization, whereas gelatinase-A and MT1-MMP may be involved in both glial invasion and angiogenesis.

**Keywords:** gliomas; gelatinase-A; gelatinase-B; MT1-MMP; in situ hybridization

The hallmarks of human malignant gliomas are their marked invasiveness and vascularity. Glioma tumour cells invade beyond the main tumour mass at diagnosis (Burger et al, 1983; Kelly et al, 1987) and render these surgically incurable. Since angiogenesis and tumour invasion have been associated with increased extracellular matrix (ECM) degradation in which the matrix metalloproteinase (MMP) family of enzymes plays a critical role, the involvement of MMPs in glioma biology is coming under increasing scrutiny.

MMPs are the principal secreted proteinases required for ECM degradation in a variety of physiological and pathological tissue remodelling processes, including wound healing, embryo implantation, tumour invasion, metastasis and angiogenesis (Woessner, 1991; Aznavoorian et al, 1993; Mignatti and Rifkin, 1993). At least 18 MMPs have been described (Pendas et al, 1997; Yong et al, 1998), which are subdivided into the collagenases, stromelysins, gelatinases and membrane-type MMPs (MT-MMPs) (Sato, 1994). Their activities are controlled at the levels of gene transcription, zymogen activation by proteolysis and inhibition of active forms by the tissue inhibitors of metalloproteinases (TIMPs) (Edwards et al, 1996). There is a wealth of evidence for an association between either deregulated MMPs and aggressive/invasive behaviour in human cancers (Davies et al, 1993; Bernhard et al, 1994; Heppner et al, 1996). This is particularly significant for gelatinase-A (MMP-2) and gelatinase-B (MMP-9) since these are critical factors in basement membrane degradation.

Gelatinase-A and gelatinase-B are controlled through distinct mechanisms. Progelatinase-A is widely expressed and is activated by a cell surface mechanism involving MT-1, -2 or -3 MMPs (Butler et al, 1997; Murphy and Knauper, 1997; Ueno et al, 1997). In contrast, progelatinase-B is controlled primarily at the level of gene expression, its transcription being activated by mitogens and inflammatory mediators (Azzam et al, 1993; Cornelius et al, 1995; Edwards et al, 1996). Furthermore, it is not activated by MT-MMPs, but is activated more promiscuously by plasmin, stromelysin-1 and gelatinase-A (Murphy and Knauper, 1997). The levels of active, rather than latent, gelatinase-A correlate best with the invasive cancer phenotype (Azzam et al, 1993; Brown et al, 1993); in breast cancer MT1-MMP is its activator (Ueno et al, 1997).

*Received 19 May 1998*
*Received 26 August 1998*
*Accepted 11 September 1998*

*Correspondence to:* PA Forsyth, Tom Baker Cancer Centre, 1331 29 St NW, Calgary, Alberta, Canada T2N 4N2

*Present address: Ontario Cancer Institute, Princess Margaret Hospital, 620 University Ave, Toronto Ontario, Canada M5G 2L7

Gelatinase-A, -B and MT1-MMP are over-expressed in glioma cell lines/surgical specimens (Apodaca et al, 1990; Nakano et al, 1993, 1995; Rao et al, 1993, 1996; Nakagawa et al, 1994, 1996; Rutka et al, 1995; Saxena et al, 1995; Sawaya et al, 1996; Uhm et al, 1996; Yamamoto et al, 1996; Forsyth et al, 1998). Immunohistochemistry shows gelatinase-A, -B, and MT1-MMP in glioma tumour cells (Costello et al, 1994; Nakagawa et al, 1994; Rao et al, 1996; Sawaya et al, 1996; Yamamoto et al, 1996). The distribution of gelatinase-A and -B may be different, however, in the tumour microvasculature. Some found both gelatinase-A and -B in endothelial cells (Costello et al, 1994; Rao et al, 1996; Sawaya et al, 1996) but others found only gelatinase-B in the tumour microvasculature (Nakagawa et al, 1994). This difference in the spatial distribution suggests the function of these in gliomas may be quite different. We hypothesize that gelatinase-B may be more important in neovascularization where it may be used by capillary endothelial cells to degrade the basement membrane and allow them to migrate towards the angiogenic stimuli; endothelial cells are known to produce MMPs and TIMPs in-vitro (Hanemaaijer et al, 1993; Cornelius et al, 1995; Lewalle et al, 1995; Zucker et al, 1995; Foda et al, 1996).

We compared the expression of gelatinase-A, -B and MT1-MMP in 47 brain tumour specimens to seven control tissues and tested the hypothesis that deregulated expression of gelatinase-A or -B, or the activator of gelatinase-A, MT1-MMP, may contribute to the aggressiveness of human gliomas. By studying these three MMPs simultaneously in a large number of the same tumour samples, we hoped to clarify their respective roles in glioma biology.

### MATERIALS AND METHODS

### Tissue collection

Procedures on patients were performed under a general anaesthetic and tumour specimens placed immediately in liquid nitrogen and stored at –80°C. The cervical lymph node containing the metastasis of patient no.1 was obtained at autopsy by dissection, snap frozen in liquid nitrogen and stored at –80°C. This study has the approval of our institutional ethics board. All patients gave signed, informed consent for their tissue to be used. The following tissues were studied: 19 glioblastoma multiforme (GBMs) (including two from the patients with extraneural metastases), one cervical metastasis from a GBM, five meningiomas (M), seven anaplastic astrocytomas (AA), five malignant oligodendrogliomas (MO), eight low grade gliomas (LGG), one clival chordoma and one spinal ependymoma; these were compared to seven controls (two normal brain samples obtained during non-brain tumour surgery and five from autopsy). All glioma tissue was obtained from regions of tumour corresponding to actively growing tumour; tumour regions containing only necrosis or relatively normal brain were not sampled.

### Zymography

This in vitro assay uses gelatin-substrate gel electrophoresis to measure the levels of metalloproteinase activity in tumour samples. Frozen tissues were pulverized in liquid nitrogen and homogenized in buffer (0.5 M Tris-HCl, pH 7.6; 0.2 M NaCl; 10 mM $CaCl_2$; 1% Triton-X100) in an Ultra-Turrax-25 homogenizer. Ten Milligrams of total protein from homogenate supernatants were electrophoresed on 10% denaturing sodium dodecyl sulphate (SDS) polyacrylamide gels containing 1 mg $ml^{-1}$ of gelatin. Gels were washed overnight in washing buffer (50 mM Tris-HCI; pH 8.0; 5 mM $CaCl_2$; 2.5% Triton-X100) and then incubated for 24 h at 37°C in the above buffer without Triton-X100 so that renaturation of enzyme could occur. Gels were stained with Coomassie blue and de-stained. Gelatinolytic activities were visualized as clear bands against a blue background. Gels were analysed by computerized densitometric scanning of the images using a Hewlett-Packard Scan Jet IIc Scanner, Deskscan II software and the NIH ‘Image’ Program. The size and intensity of each band were determined and its area plotted on graphs.

### RT-PCR

#### *RNA preparation*

Total RNA was extracted from cells by the acid guanidinium isothiocyanate method. The final RNA concentrations were determined by absorption using a GeneQuant spectrophotometer (Pharmacia).

#### *Reverse transcription reactions*

Each 20 μl cDNA synthesis reaction contained 1 μg of total RNA, 1 × PCR buffer (10 mM Tris-HCl; pH 9.0; 50 mM KCl; 1.5 mM magnesium chloride), 1 mM each of deoxynucleotide triphosphates (dATP, dGTP, dCTP, and dTTP), 20 units placental ribonuclease inhibitor (RNAguard, Pharmacia), 200 units of MuLV-reverse transcriptase (RT) (Bethesda Research Laboratories) and 100 pmol of random hexamer oligodeoxynucleotides (Pharmacia). Reaction mixtures were preincubated 10 min at 21°C prior to cDNA synthesis. The reverse transcription reactions were carried out for 50 min at 42°C and were heated to 95°C for 5 min to terminate the reaction. Samples were cooled to 4°C or stored at –20°C until use.

#### *PCR reactions*

Multiplex PCRs were performed in 50 μl reaction volumes. Each reaction contained 2 μl of RT reaction product as template DNA (corresponding to cDNA synthesized from 100 ng of total RNA), 1 × PCR buffer, 80 μm of each deoxynucleotide (in addition to the dNTP left over from the RT reaction, resulting in a final concentration of approximately 180 μM) and 20 pmol each of 5′ and 3′ target primers. Two units of *Taq* DNA polymerase (Gibco-BRL) were added to each tube during the first denaturation step (‘hot start’) and equal aliquots (20 pmol) of GAPDH primer sets were added at the appropriate cycle number by the ‘primer dropping’ method (Wong et al, 1994). Each PCR cycle consisted of a heat-denaturation step at 94°C for 1 min, a primer-annealing step at 55°C for 30 s, and a polymerization step at 72°C for 1 min. PCR amplifications were performed in a Temp-Tronic Thermal Cycler (Barnstead/Thermolyne). Aliquots of PCR reaction products (approximately 10 μl) equalized to give equivalent signals from the internal control mRNA (GAPDH) were electrophoresed through 2% agarose gels containing 0.2 mg of ethidium bromide. To allow quantification of RT-PCR data, an initial ‘cycle test’ was performed for each sample and primer set to determine the appropriate number of cycles required for detection of amplification products while remaining in the exponential phase of PCR. For gelatinase-A, gelatinase-B and MT1-MMP amplifications, the operative cycle numbers were 29, 33 and 30 respectively. GAPDH primers were added to the last 23 cycles. Gels were illuminated

© Cancer Research Campaign 1999

with UV light, photographed using Polaroid film, and analysed by computerized densitometric scanning as described above. The intensities of the ethidium bromide fluorescence signals were determined from the area under the curve for each peak and the data were plotted on graphs.

The following primers were employed:

| | |
|---|---|
| Gelatinase-A | 5′-primer=5′-GCGGATCCAGCGCCCAGAGAGACAC |
| | 3′-primer=3′-TTAAGCTTCCACTCCGGGCAGGATT |
| Gelatinase-B | 5′-primer= 5′-TGGACGATGCCTGCAACGTG |
| | 3′-primer= 5′-GTCGTGCGTGTCCAAAGGCA |
| MT1-MMP | 5′-primer= 5′-GCCCATTGGCCAGTTCTGGCCGG |
| | 3′-primer= 5′-CCTCGTCCACCTCAATGATGATC |
| GAPDH | 5′-primer= 5′-CGGAGTCAACGGATTTGGTCGTAT |
| | 3′-primer= 5′-AGCCTTCTCCATGGTGGTGAAGAC |

The size of the amplification products were 473 bp for gelatinase-A, 454 bp for gelatinase-B, 548 bp for MT1-MMP and 306 bp for GAPDH respectively.

### In situ hybridization for gelatinase-A, -B and MT1-MMP mRNA

A 348 bp *Bg*l-*Bam*HI fragment of human gelatinase-A (sequence corresponding to 1404–1752) was obtained by RT-PCR from human Hs68 cell RNA and cloned into pBluescript KS- (Stratagene). The construction of the 390 bp gelatinase-B probe has been described previously (Urbanski et al, 1992). The MT1-MMP probe was a 420 bp region encompassing the pro-domain and part of the catalytic domain, cloned in pBluescript SK+ based on the sequence of Sato et al (1994) and generously provided by Dr Suneel Apte, Cleveland Clinic Foundation, Cleveland, OH, USA. For gelatinase-A, antisense riboprobe was generated with T3 polymerase from template linearized with *Xba*I, and sense riboprobe was produced with T7 polymerase and *Bam*HI-cut plasmid. Corresponding probes for gelatinase-B involved: T7 polymerase and *Hin*dIII-cut template (antisense), T3 polymerase and *Not*I-cut template (sense). For MT1-MMP, we used T3 polymerase and *Sal*I-cut template (anti-sense); T7 polymerase and *Eco*RI-cut plasmid (sense). Riboprobes were prepared and labelled with digoxygenin (DIG)-labelled-UTP (Boehringer Mannheim, Laval, Quebec, Canada) following the manufacturer's instructions. Confirmation of sense and antisense riboprobe was confirmed by Northern blot analysis. Antisense probes but not sense probes, detected a single band of the appropriate size for all three genes.

In situ hybridization was performed as described previously (Harvey et al, 1995; Leco et al, 1997). Briefly, 4-μm paraffin-embedded brain sections were dewaxed in xylene and rehydrated through a series of graded ethanols. Sections were treated with proteinase K (20 μg $ml^{-1}$), acetylated and then prehybridized in buffer containing 50% formamide, 5 × SSPE, 1 × Denhardt's solution, 20 mM DTT for 6 h at 50°C. Hybridization was done at 60°C overnight in the same buffer to which 20 ng $ml^{-1}$ of probe and 8 μg $ml^{-1}$ of *Escherichia coli* tRNA were added. After hybridization, sections were then washed once in 2 × SSC at 37°C, treated with 20 mg $ml^{-1}$ RNase A at 37°C, washed once in 2 × SSC at 50°C, once in 50% formamide at 50°C, twice in 2 × SSC at 50°C and once in 0.5 × SSC at 50°C; all for 30 min each time. Following blocking, sections were incubated in a 1:1000 dilution of sheep anti-DIG-alkaline phosphatase conjugated antibody (Boehringer Mannheim), for 4 h at room temperature. After extensive washing, NBT/BCIP chromogens were applied to sections and colour was developed in the dark until the desired intensity was obtained. The reaction was terminated by placing sections in 20 mM Tris-HCl, pH 7.5, 10 mM EDTA. Sections were then dipped briefly in water, counter-stained for 3 min in 0.02% fast green, washed for 1 min in water and mounted with Advantage aqueous mounting medium (Accurate Chemical). The slides were photographed on Kodak Royal Gold 35 mm film using a Zeiss photomicroscope II under bright-field illumination.

### Statistical analysis

The quantitative expression of gelatinase-A and -B in terms of lysis per mg of protein from the zymograms, or in terms of their transcript intensity for gelatinase-A, -B and MT1-MMP from RT-PCR were compared using the Kruskal–Wallis test. These quantitative expressions were correlated with glial malignancy (normal low grade versus malignant glioma versus GBM) using Spearman's rank correlation coefficient. Data for meningiomas and other brain tumours were plotted but not analysed statistically.

### RESULTS

### Detection of gelatinase-A, -B, and MT1-MMP mRNA by RT-PCR analysis

Gelatinase-A, -B and MT1-MMP mRNA expression was evaluated by RT-PCR and compared with frozen samples of histologically defined normal brain tissue and brain tumours (Figure 1). Gelatinase-A, -B and MT1-MMP RNA expression was very low in the normal samples. PCR amplification of cDNA prepared from frozen samples defined histologically as low grade glioma (oligodendroglioma, astrocytoma, oligo-astrocytoma, pilocytic

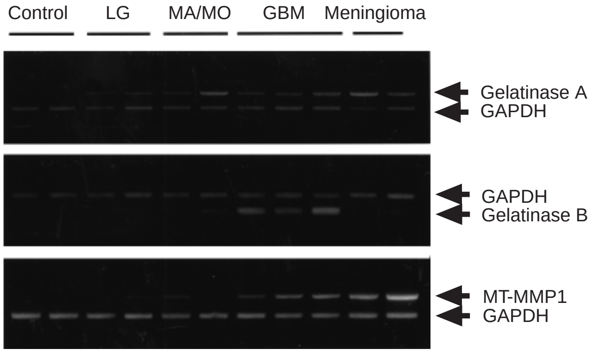

**Figure 1** Representative examples of expression of gelatinase-A, -B and MT1-MMP mRNA using RT-PCR in human brain tumour and normal brain tissue. Two control samples have low levels of transcripts of all three genes. Tumour levels of gelatinase-A and MT1-MMP were higher than in normal tissues and varied somewhat but did not strongly correlate with tumour malignancy. Gelatinase-B RNA levels were also variable but were much higher in GBMs than in low grade gliomas. Higher levels of gelatinase-A and MT1-MMP than gelatinase-B RNAs were seen in meningiomas. Abbreviations for all Figures: AA = anaplastic astrocytoma; AO = anaplastic oligodendroglioma; CLN-1 = cervical lymph node metastasis; ENM = extraneural metastasis; GBM = glioblastoma multiforme; GBM-1, -2 = first and second GBM patient with extramural metastases; LGG = low grade glioma; MO/MA = malignant oligodendroglioma or malignant astrocytoma; N1-7 = normal tissue; Other = 1 clival chordoma and 1 spinal ependymoma; RA = reactive astrocytes

 *© Cancer Research Campaign 1999*

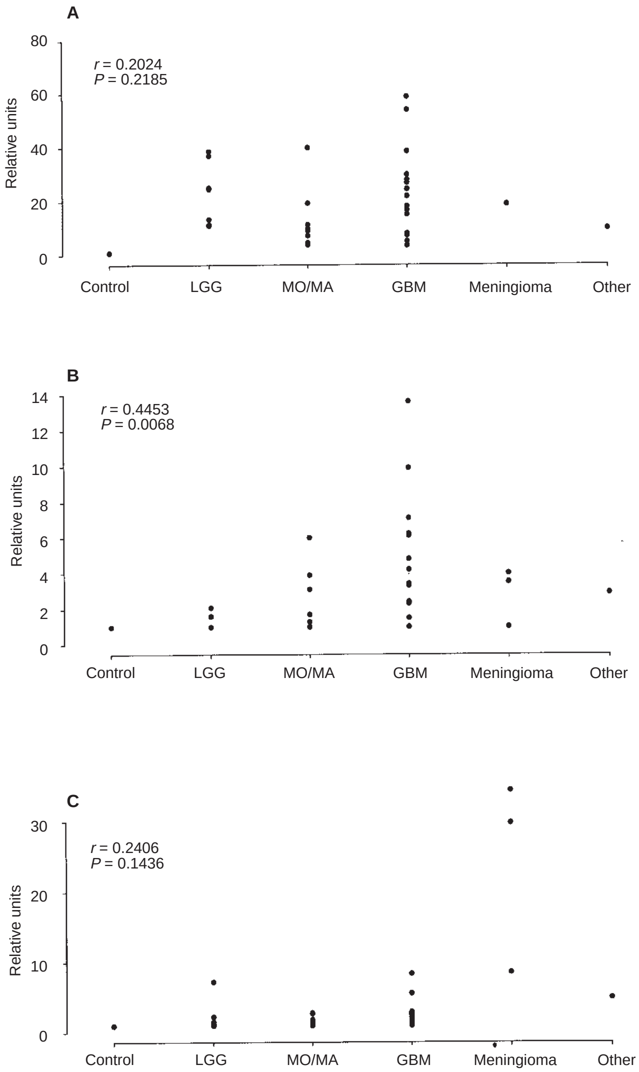

**Figure 2** Quantification of gelatinase-A, -B, and MT1-MMP mRNA in human brain tumours. The relative intensities of bands using densitometry were compared to the average of two control samples, and plotted in arbitrary units. Correlations between normal tissues and the various grades of gliomas were determined for gelatinase-A (**A**), gelatinase-B (**B**), and MT1-MMP (**C**)

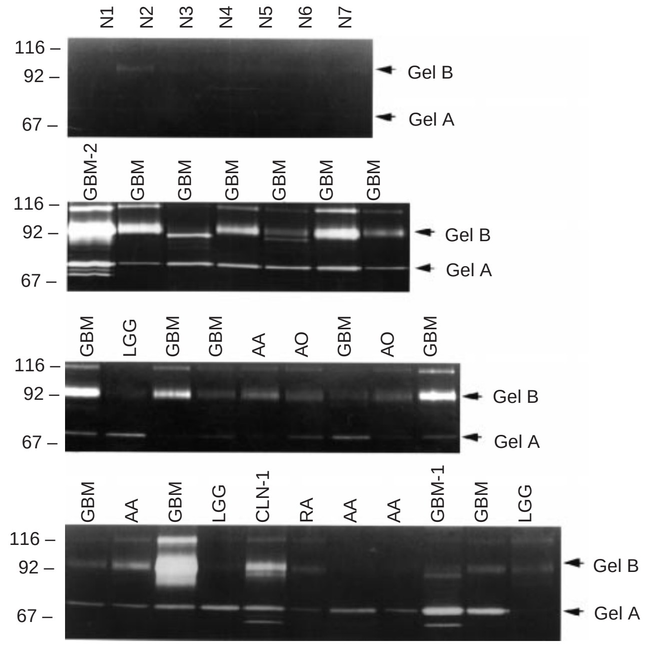

**Figure 3** Zymographic analysis of gelatinase-A and -B activities in human brain tumours and normal brain. Progelatinase-A and progelatinase-B were detected as prominent bands of activity at 72 kDa and 92 kDa respectively. Minor, faster migrating forms that may be activated species were seen in a few specimens. Higher molecular weight forms which may correspond to complexes of pro-gelatinases with TIMPs were also observed

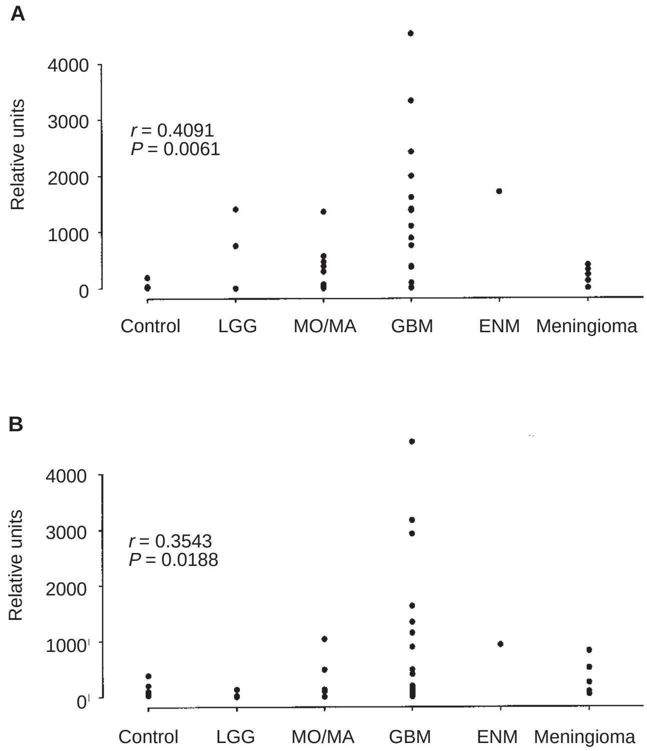

**Figure 4** Average zymographic lysis values for brain tumours and normal brain tissue. The amount of lytic clearing (calculated as densitometric units/microgram) from Figure 3 was quantified as described in Methods for gelatinase-A (**A**) and gelatinase-B (**B**)

astrocytoma), malignant gliomas (anaplastic astrocytomas or malignant oligodendroglioma), glioblastoma, meningioma, or other (clival chordoma or spinal ependymoma) demonstrated the presence of gelatinase-A, -B and MT1-MMP transcripts; these were consistently over-expressed in tumour samples compared to normal tissues. Expression of gelatinase-A and MT1-MMP was highly variable and did not correlate with degree of glioma malignancy ($r$ = 0.2024 Spearman's rank correlation coefficient, $P$ = 0.2185 and $r$ = 0.2406 Spearman's rank correlation coefficient, $P$ = 0.1436 respectively) (Figure 2). In contrast, gelatinase-B RNA levels were also variable but correlated more strongly with glioma malignancy with higher levels seen in higher grades ($r$ = 0.4453 Spearman's rank correlation coefficient, $P$ = 0.0068).

© Cancer Research Campaign 1999 

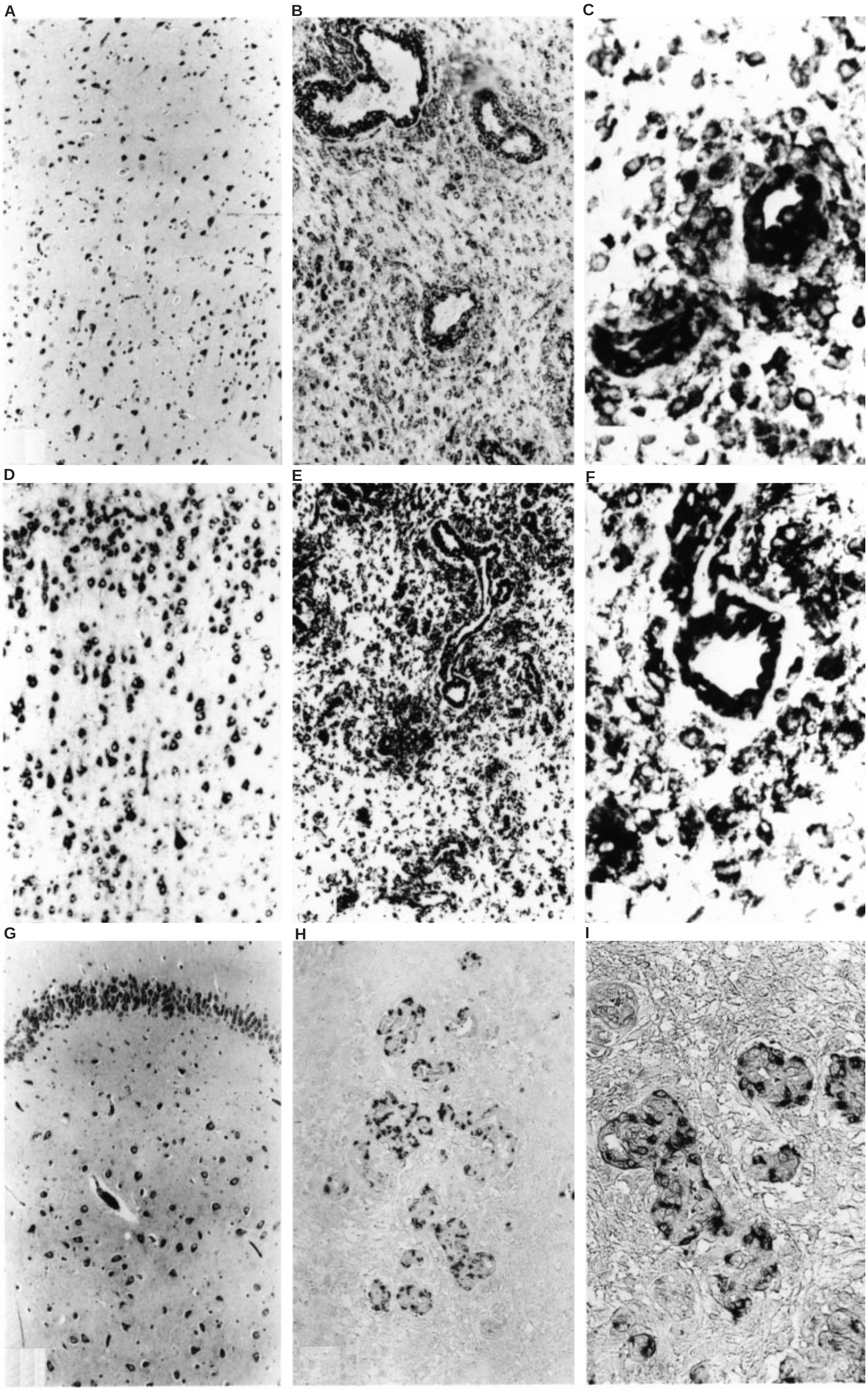

**Figure 5** Paraffin sections of GBMs stained using in situ hybridization for gelatinase-A (**A**, **B**, **C**), MT1-MMP (**D**, **E**, **F**), and gelatinase-B (**G**, **H**, **I**). Gelatinase-A: (**A**) Normal cortex shows signal in neurons, less in glia, and blood vessels (100×). (**B**) The tumour margin of a GBM shows diffusely positive staining; signal is seen in tumour cells and blood vessels (100×). (**C**) A higher power (400×) picture of the GBM seen in panel **B** which shows diffusely positive staining even in the centre of the tumour. MT1-MMP: The distribution of MT1-MMP mRNA is similar to gelatinase-A. (**D**) Normal cortex shows MT1-MMP signal is present in neurons and glia. (**E**) Both tumour cells and blood vessels show staining for MT1-MMP at lower (100×) and higher powers (400×) (**F**) both at the centre and tumour margins in this GBM. Gelatinase-B: (**G**) Normal hippocampal cortex shows gelatinase-B is localized to neurons (the cytoplasm more than nucleus) and glia. (**H**) At the tumour margin in this GBM the gelatinase-B is localized to perivascular cells (100×). There was no signal seen at the tumour centre but only at the proliferating margins. (**I**) A higher magnification (400×) of panel **H** showing expression is restricted to cells that are not immediately adjacent to the lumens of these small blood vessels which may be smooth muscle cells or pericytes

 *© Cancer Research Campaign 1999*

Though few meningiomas were studied, there were very high levels of gelatinase-A and MT1-MMP expression. Of the four tumour specimens showing the highest levels of gelatinase-B expression, two were from patients whose tumours subsequently had extraneural metastases and a third from a patient whose GBM eroded through his skull (Table 1); both are very rare events.

### Zymographic analysis

The activities of gelatinase-A and -B in human brain tumours and normal tissue were analysed by gelatin zymography. The latent form of gelatinase-A (72 kDa) was detected in almost every tissue extract. Low levels of both gelatinase-A and -B activity were found in the control specimens and these were similar to the activities seen in low grade gliomas. Progelatinase-A levels were highly variable within each tumour type and correlated with the degree of tumour malignancy ($r = 0.4091$ Spearman's rank correlation coefficient, $P = 0.0061$) (Figures 3 and 4). We found active gelatinase-A only in the two patients who had extraneural metastases from their GBM and in the cervical extraneural metastasis itself in one patient. Progelatinase-A was high or intermediate in meningiomas.

For gelatinase-B there was a trend for higher levels of activity to be seen in more malignant gliomas; levels were highest in GBMs, and consistently higher than activities in controls or low grade gliomas ($r = 0.3543$ Spearman's rank correlation coefficient, $P = 0.018$). Gelatinase-B activity was much lower than gelatinase-A in meningiomas (see Figure 4).

### Localization of gelatinase-A, -B and MT1-MMP mRNA in normal brain and human brain tumour tissues

The identity of cells expressing gelatinase-A, -B and MT1-MMP was determined by in situ analysis of paraffin-embedded sections. As observed in our analysis above, gelatinase-A and MT1-MMP were expressed in very low, but detectable, levels in normal brain tissue, most prominently in neurons with much less signal in glial cells and blood vessels. In tumours, gelatinase-A and MT1-MMP were expressed in the tumour cells and in many cell types in the surrounding stroma, including neurons, glia and blood vessels (Figure 5A–F). Gelatinase-B expression (Figure 5G–I) was present at very low levels in normal tissues (principally neurons in the hippocampus; Figure 5G and data not shown) but in the tumours it was largely restricted to regions in blood vessels that were undergoing endovascular proliferation at the infiltrating border of the tumour (Figure 5H, I). For most tumours studied there was no detectable staining of gelatinase-B in tumour cells themselves, in the rest of the surrounding stroma, or in blood vessels in other areas that were not proliferating, such as the centre of the tumour. However, in three other patients the pattern of expression was more diffuse and gelatinase-B expression was present in all cell types in the tumour.

These in situ hybridization data can be summarized as follows:

1. Expression of gelatinase-A and MT1-MMP showed close correspondence. Both were present in neurons in normal brain, and in both tumour and stromal cell types in tumour. Though in situ hybridization is not quantitative, signals were weak in normal brain, but usually strong in tumours, indicating a general concordance with the RT-PCR data in Figure 1.
2. Gelatinase-B was also weakly expressed in neurons in normal brain, but in contrast to gelatinase-A and MT1-MMP its expression in tumours was most evident in proliferating blood vessels at tumour margins. Some tumours showed endothelial cell positivity for gelatinase-B transcripts, while the example shown in Figure 5 had pronounced expression in surrounding cell types which are likely vascular smooth muscle cells or pericytes.

### DISCUSSION

The significance of the present work rests on several key points. Firstly, this is the largest study undertaken to date on primary human brain tumour specimens to assess the role of MMPs, and the first to simultaneously evaluate the contributions of gelatinase-A, gelatinase-B and MT1-MMP in a common set of brain tumour specimens. Secondly, our results show that although all three MMPs are likely connected in some way with malignant behaviour in gliomas, gelatinase-B is the most closely correlated with tumour grade. Thirdly, we provide the first in situ hybridization data to localize the cellular origins of MMP expression in gliomas. These studies lead to the notion that gelatinase-B is primarily involved in tumour neovascularization, wheras the widespread and similar localization of gelatinase-A and MT1-MMP mRNAs suggests involvement in both invasion and angiogenesis.

Our in situ hybridization results show that the transcripts of gelatinase-A, -B and MT1-MMP have different cellular origins. Gelatinase-A and MT1-MMP were both expressed by many cell types in gliomas, including the microvasculature and the tumour cells themselves (Costello et al, 1994; Yamamoto et al, 1996; Sawaya et al, 1996). We observed expression of both gelatinase-A and MT1-MMP RNAs mostly in neurons in normal tissue, with low signals from blood vessel elements. This suggests that an important contribution of the increased expression of gelatinase-A and MT1-MMP in malignant gliomas compared to normal brain relates to neovascularization at tumour margins and blood vessel expansion deep within the tumour. The other component of increased gelatinase-A and MT1-MMP expression is the tumour cells themselves, which confirms other immunolocalization work (Yamamoto et al, 1996; Sawaya et al, 1996).

The restricted perivascular localization of gelatinase-B expression by in situ hybridization agrees with immunodetection of gelatinase-B (Rao et al, 1996; Nakagawa et al, 1994). In some tumours we observed gelatinase-B in endothelial cells and tumour cells but in others we found expression confined to cells lying deep to the vessel endothelium. These are most likely pericytes and/or smooth muscle cells and angiogenesis involves cooperation between these (Folkman, 1971; Hirschi and D'Amore, 1996). Pericyte/vascular smooth muscle cell (VSMC) proliferation has been suggested to be an early event in microvascular proliferation in GBMs (Wesseling et al, 1995) where it may be critical for new vessel formation (Wesseling et al, 1995; Haddad et al, 1992). Gelatinase-B expression may be associated with early rapid pericyte proliferation during angiogenesis and later during vessel growth expression is taken over by endothelial cells themselves.

Several changes in gene expression in the tumour and the surrounding vasculature must occur to induce neovascularization (Hanahan & Folkman, 1996). Evidence that MMP activity is required for neovascularization includes observations that TIMPs and synthetic MMP inhibitors block angiogenesis in a number of experimental systems (Rosenthal et al, 1994; Johnson et al, 1994;

© Cancer Research Campaign 1999

Schnaper et al, 1993; Taraboletti et al, 1995); though some (Thorgeirsson et al, 1996) suggest the anti-angiogenic activities of TIMP-1 may not be mediated by its anti-metalloproteinase effects. Inhibition of MMP activity using a synthetic inhibitor GM6001 has also been shown to block smooth muscle cell migration in vivo which may also contribute to the antiangiogenic actions of these molecules (Bendeck et al, 1996). Our demonstration that gelatinase-B expression is localized to the vasculature at the proliferating borders in gliomas provides further support for the importance of MMPs in angiogenesis.

We provide further support for the notion that in gliomas the MMPs gelatinase-A and MT1-MMP are both produced and used by the tumour cells themselves (Rao et al, 1996; Costello et al, 1994; Nakagawa et al, 1994; Yamamoto et al, 1996; Sawaya et al, 1996). While it would seem intuitively obvious that tumour cells would produce MMPs in many systemic cancers it is the surrounding non-tumoural stromal cells that produce them (Heppner et al, 1996; Pyke et al, 1993; Poulsom et al, 1993). One explanation for the differences in cellular expression of MMPs in gliomas versus in systemic cancers may be the nature of the specialized 'stroma' in the brain. Systemic cancers are often confined by tough basement membranes and collagen-rich tissue whereas the brain's ECM is composed chiefly of hyaluronan and proteoglycans (Giese and Westphal 1996). In addition to posing a less formidable barrier to invasion the brain's stromal environment may regulate proteinase expression in glioma cells.

Malignant gliomas, and GBMs in particular, are both highly invasive and vascular tumours and our results suggest that both of these processes depend in part on the increased expression of gelatinase-A, MT1-MMP and gelatinase-B. Changes in the expression patterns of several MMPs might be important in different human brain tumours at different times in their malignant progression. For example, gelatinase-A may be expressed at an early stage in tumorigenesis and support glioma invasion but gelatinase-B may be employed at later stages in malignant progression to help provide and maintain tumour vasculature. The mechanisms underlying the upregulation of these MMPs in gliomas are unknown and need to be better understood. This would provide important information about the regulatory pathways controlling glioma invasion and angiogenesis and suggest appropriate targets for clinical therapies.

### ACKNOWLEDGEMENTS

We thank Dr JG Cairncross for his encouragement and support and Ms Eve Lee for expert assistance in manuscript preparation. We thank Dr David Ramsay and the Canadian Brain Tumor Tissue Bank for some glioma specimens. Supported by: Alberta Cancer Board (DRE, PF, RNJ, NBR), Hugo and Edith Weiss Memorial Endowment, and Harry W Higgins Endowment (PF) and Foothills Hospital Foundation (PF, RNJ). TDL holds a studentship from the Medical Research Council of Canada and the Alberta Heritage Foundation for Medical Research (AHFMR). KJL was supported by a AHFMR postdoctoral fellowship. DRE is a Senior Scholar of the AHFMR.

### REFERENCES

Apodaca G, Rutka JT, Bouhana K, Berens ME, Giblin JR, Rosenblum ML, McKerrow JH and Banda MJ (1990) Expression of metalloproteinase and metalloproteinase inhibitors by fetal astrocytes and glioma cells. *Cancer Res* **50**: 2322–2329

Aznavoorian S, Murphy AN, Stetler-Stevenson WG and Liotta LA (1993) Molecular aspects of tumor cell invasion and metastasis. *Cancer* **71**: 1368–1383

Azzam HS, Arand G, Lippman ME and Thompson WE (1993) Association of MMP-2 activation potential with metastatic progression in human breast cancer cell lines independent of MMP-2 production. *J Natl Cancer Inst* **85**: 1758–1754

Bendeck MP, Irvin C and Reidy MA (1996) Inhibition of matrix metalloproteinase activity inhibits smooth muscle cell migration but not neointimal thickening after arterial injury. *Circ Res* **78**: 38–43

Bernhard EJ, Gruber SB and Muschel RJ (1994) Direct evidence linking expression of matrix metalloproteinase 9 (92 kDa gelatinase/collagenase) to the metastatic phenotype in transformed rat cells. *Proc Natl Acad Sci USA* **91**: 4293–4297

Brown PD, Bloxidge RE, Stuart NS, Gatter KC and Carmichael J (1993) Association between expression of activated 72-kilodalton gelatinase and tumor spread in non-small cell lung carcinoma. *J Natl Cancer Inst* **85**: 574–579

Burger PC, Dubois PJ, Schold SC Jr, Smith KR Jr, Odom GL, Crafts DC and Giangaspero F (1983) Computerized tomographic and pathologic studies of the untreated, quiescent, and recurrent glioblastoma multiforme. *J Neurosurg* **58**: 159–169

Butler GS, Will H, Atkinson SJ and Murphy G (1997) Membrane-type-2 matrix metalloproteinase can initiate the processing of progelatinase A and is regulated by the tissue inhibitors of metalloproteinases. *Eur J Biochem* **244**: 653–657

Cornelius LA, Nehring LC, Roby JD, Parks WC and Welgus HG (1995) Human dermal microvascular endothelial cells produce matrix metalloproteinases in response to angiogenic factors and migration. *J Invest Dermatol* **105**: 170–176

Costello PC, Del Maestro RF and Stetler-Stevenson WG (1994) Gelatinase A expression in human malignant gliomas. *Ann NY Acad Sci* **732**: 450–452

Davies B, Waxman J, Wasan H, Abel P, Williams G, Krausz T, Neal D, Thomas D, Hanby A and Balkwill F (1993) Levels of matrix metalloproteinases in bladder cancer correlate with tumor grade and invasion. *Cancer Res* **53**: 5365–5369

Edwards DR, Beaudry PP, Laing TD, Kowal V, Leco KJ and Lim MS (1996) The roles of tissue inhibitors of metalloproteinases in tissue remodelling and cell growth. *Int J Obesity* **20**: S9–S15

Foda HD, George S, Conner C, Drews M, Tompkins DC and Zucker S (1996) Activation of human umbilical vein endothelial cell progelatinase A by phorbol myristate acetate: a protein kinase C-dependent mechanism involving a membrane-type matrix metalloproteinase. *Lab Invest* **74**: 538–545

Folkman J (1971) Tumor angiogenesis: therapeutic implications. *N Engl J Med* **285**: 1182–1186

Forsyth PA, Dickinson-Laing T, Gibson AW, Rewcastle NB, Brasher P, Sutherland G, Johnston RN and Edwards DR (1998) High levels of gelatinase-B and active gelatinase-A in metastatic glioblastoma. *J Neurooncol* **36**: 21–29

Giese A and Westphal M (1996) Glioma invasion in the central nervous system. *Neurosurgery* **39**: 235–252

Haddad SF, Moore SA, Schelper RL and Goeken JA (1992) Vascular smooth muscle hyperplasia underlies the formation of glomeruloid vascular structures of glioblastoma multiforme. *J Neuropathol Exp Neurol* **51**: 488–492

Hanahan D and Folkman J (1996) Patterns and emerging mechanisms of the angiogenic switch during tumorigenesis. *Cell* **86**: 353–364

Hanemaaijer R, Koolwijk, Le Clerc QL, De Vree WJA and Van Hinsbergh VWM (1993) Regulation of matrix metalloproteinase expression in human vein and microvascular endothelial cells. Effects of tumour necrosis factor $\alpha$, interleukin 1 and phorbol ester. *Biochem J* **296**: 803–809

Heppner KJ, Matrisian LM, Jensen RA and Rodgers WH (1996) Expression of most MMP family members in breast cancer represents a tumor-induced host response. *Am J Pathol* **149**: 273–282

Hirschi KK and D'Amore P (1996) Pericytes in the microvasculature. *Cardiovasc Res* **32**: 687–698

Johnson MD, Kim HR, Chesler L, Tsao Wu-G, Bouck N and Polverini PJ (1994) Inhibition of angiogenesis by TIMP. *J Cell Physiol* **160**: 194–202

Kelly PJ, Daumas-Duport C, Scheithauer BW, Kall BA and Kispert DB (1987) Stereotactic histologic correlations of computed tomography and magnetic resonance imaging-defined abnormalities in patients with glial neoplasms. *Mayo Clinic Proc* **62**: 450–459

Leco KJ, Apte SS, Taniguchi GT, Hawkes SP, Khokha R, Schultz GA and Edwards DR (1997) Murine tissue inhibitor of metalloproteinases-4 (TIMP-4): cDNA isolation and expression in adult mouse tissues. *FEBS Letts* **401**: 213–217

Lewalle JM, Munaut C, Pichot B, Cataldo D, Baramova E and Foidart JM (1995) Plasma membrane-dependent activation of gelatinase A in human vascular endothelial cells. *J Cell Physiol* **165**: 475–483

Mignatti P and Rifkin DB (1993) Biology and chemistry of proteinases in tumor invasion. *Physiol Rev* **73**: 161–185

 *© Cancer Research Campaign 1999*

Murphy G and Knauper V (1997) Relating matrix metalloproteinase structure to function: Why the ‘Hemopexin’ domain? *Matrix Biol* **15**: 511–518

Nakagawa T, Kubota T, Kabuto M, Sato K, Kawano H, Hayakawa T and Okada Y (1994) Production of matrix metalloproteinases and tissue inhibitor of metalloproteinases-1 by human brain tumors. *J Neurosurg* **81**: 69–77

Nakagawa T, Kubota T, Kabuto M, Fujimoto N and Okada Y (1996) Secretion of matrix metalloproteinases-2 (72 kD gelatinase/type IV collagenase = gelatinase A) by malignant human glioma cell lines: implications for the growth and cellular invasion of the extracellular matrix. *J Neurooncol* **28**: 13–24

Nakano A, Tani E, Miyazaki K, Yamamoto Y and Furuyama J (1995) Matrix metalloproteinases and tissue inhibitors of metalloproteinases in human gliomas. *J Neurosurg* **83**: 298–307

Nakano A, Tani E, Miyazaki K, Furuyama J and Matsumoto T (1993) Expressions of matrilysin and stromelysin in human glioma cells. *Biochem Biophys Res Commun* **192**: 999–1003

Pendas AM, Knauper V, Puente XS, Llano E, Mattei M-G, Apte S, Murphy G and Lopez-Otin C (1997) Identification and characterization of a novel human matrix metalloproteinase and unique structural characteristics, chromosomal location and tissue distribution. *J Biol Chem* **272**: 4281–4286

Poulsom R, Hanby AM, Pignatelli M, Jeffrey RE, Longcroft JM, Rogers L and Stamp GWH (1993) Expression of gelatinase-A and TIMP-2 mRNAs in desmoplastic fibroblasts in both mammary carcinomas and basal cell carcinomas of the skin *J Clin Pathol* **46**: 429–436

Puente XS, Pendas AM, Llano E, Velasco G and Lopez-Otin C (1996) Molecular cloning of a novel membrane-type matrix metalloproteinase from a human breast carcinoma. *Cancer Res* **56**: 944–949

Pyke C, Ralfkiaer E, Tryggvason K and Dano K (1993) Messenger RNA for two types of type IV collagenases is located in stromal cells in human colon cancer. *Am J Pathol* **142**: 359–365

Rao JS, Steck PA, Mohanam S, Stetler-Stevenson WG, Liotta LA and Sawaya R (1993) Elevated levels of Mr 92000 type IV collagenase in human brain tumors. *Cancer Res* **53**: 2208–2211

Rao JS, Yamamoto M, Mohaman S, Gokaslan ZL, Stetler-Stevenson WG, Rao VH, Fuller GN, Liotta LA, Nicolson GL and Sawaya RE (1996) Expression and localization of 92 kDa type IV collagenase/gelatinase B (MMP-9) in human gliomas. *Clin Exp Metastasis* **14**: 12–18

Rosenthal RA, Moses MA, Shintani Y, Megyesi JF, Langer R and Folkman J (1994) Purification and characterization of two collagenase inhibitors from mouse sarcoma 180 conditioned medium. *J Cell Biochem* **56**: 97–105

Rutka JT, Matsuzawa K, Hubbard SL et al (1995) Expression of TIMP-1, TIMP-2, 72- and 92-kDa type IV collagenase transcripts in human astrocytoma cell lines: correlation with astrocytoma cell invasiveness. *Int J Oncol* **6**: 877–884

Sato H, Takino T, Okada Y, Cao J, Shinagawa A, Yamamoto E and Seiki M (1994) A matrix metalloproteinase expressed on the surface of invasive tumour cells. *Nature* **370**: 61–65

Sawaya RE, Yamamoto M, Gokaslan ZL, Wang SW, Mohanam S, Fuller GN, McCutcheon IE, Stetler-Stevenson WG, Nicolson GL and Rao JS (1996) Expression and localization of 72 kDa type IV collagenase (MMP-2) in human malignant gliomas in vivo. *Clin Exp Metastasis* **14**: 35–42

Saxena A, Robertson JT and Kufta C (1995) Increased expression of gelatinase A and TIMP-2 in primary human glioblastomas. *Int J Oncol* **7**: 469–473

Schnaper HW, Grant DS, Stetler-Stevenson WG, Fridman R, D’Orazi C, Murphy AN, Bird RE, Hoythya M, Fuerst TR, French DL, Quigley JP and Kleinman HK (1993) Type IV collagenase(s) and TIMPs modulate endothelial cell morphogenesis in vitro. *J Cell Physiol* **156**: 235–246

Taraboletti G, Garofalo A, Belotti D, Drudis T, Borsotti P, Scanziani E, Brown PD and Giavazzi R (1995) Inhibition of angiogenesis and murine hemangioma growth by Batimasatat, a synthetic inhibitor of matrix metalloproteinases. *J Natl Cancer Inst* **87**: 293–298

Thorgeirsson UP, Yoshiji H, Sinha CC and Gomez DE (1996) Breast cancer; tumor neovasculature and the effect of TIMP-1 on angiogenesis. *In vivo* **10**: 137–144

Ueno H, Nakamura H, Inoue M, Imai K, Noguchi M, Sato H, Seiki M and Okada Y (1997) Expression and tissue localization of membrane-types 1, 2, and 3 matrix metalloproteinases in human invasive breast carcinomas. *Cancer Res* **57**: 2055–2060

Uhm JH, Dooley NP, Villemure J-G and Yong VW (1996) Glioma invasion in vitro: regulation by matrix metalloproteinase-2 and protein kinase C. *Clin Exp Metastasis* **14**: 421–433

Urbanski SJ, Edwards DR, Maitland A, Leco KJ, Watson A and Kossakowska AE (1992) Expression of metalloproteinases and their inhibitors in primary pulmonary carcinomas. *Br J Cancer* **66**: 1188–1194

Wesseling P, Schlingemann RO, Rietveld FJR, Link M, Burger PC and Ruiter DJ (1995) Early and extensive contribution of pericytes/vascular smooth muscle cells to microvascular proliferation in glioblastoma multiforme: an immuno-light and immuno-electron microscopic study. *J Neuropathol Exp Neurol* **54**: 304–310

Woessner JF (1991) Matrix metalloproteinases and their inhibitors in connective tissue remodelling. *FASEB J* **5**: 2145–2154

Wong H, Anderson WD, Cheng T and Riabowol KT (1994) Monitoring mRNA expression by polymerase chain reaction: the ‘primer-dropping’ method. *Anal Biochem* **223**: 251–258

Yamamoto M, Mohanam S, Sawaya R, Fuller GN, Seiki M, Sato H, Gokaslan ZL, Liotta LA, Nicolson GL and Rao JS (1996) Differential expression of membrane-type matrix metalloproteinase and its correlation with gelatinase-A activation in human malignant brain tumors in vivo and in vitro. *Cancer Res* **56**: 384–392

Yong VW, Krekowski CA, Forsyth PA, Bell R and Edwards D (1998) Matrix Metalloproteinases and diseases of the CNS. *Trends Neurosci* **21**: 75–80

Zucker S, Cponner C, DiMassmo BI, Ende H, Drews M, Seiki M and Bahou WF (1995) Thrombin induces the activation of progelatinase A in vascular endothelial cells. *J Biol Chem* **270**: 23730–23738

*© Cancer Research Campaign 1999*
